# Remarks upon Making Gold Plate and Solder

**Published:** 1853-01

**Authors:** B. Wood

**Affiliations:** Dentist.


### ARTICLE XVIII.

### *Remarks upon making Gold Plate and Solder.* By B. WOOD, M. D., Dentist.

MESSRS. EDITORS:—Having recently made some experiments in the way of preparing gold plate and solder, I take the liberty of submitting the result, with the hope that it may elicit further information from some of your readers, who may be more conversant with the working of metals.

*Gold Plate.*—The ordinary alloy of eighteen to twenty, or even twenty-one carats, for gold plate, is liable to objection. In some mouths it will tarnish, and even corrode. A plate not liable to this, may be made by alloying pure gold with a small proportion of platinum; and for the past few years, this expedient has been resorted to by dentists. Still, the plate thus made is objectionable. If enough platinum is used to give the necessary elasticity, &c., it impairs the color, as well as the susceptibility of receiving a good polish. Besides, the peculiar gray and almost dirty hue is apt to suggest the suspicion that the plate is base. But a few days ago, I was called upon to testify, for a worthy professional brother, to the fineness of the gold used in an artificial set of teeth, the plate of which was alloyed in this way. It appears the patient had exhibited the work to a quack dentist, who, doubtless, because the gold did not, in point of *color*, compare well with his of sixteen or eighteen carat (alloyed with copper or silver) succeeded in making the impression that it was very impure.

The object of my experiment was to obtain a plate which, while as fine as possible, should possess the requisite hardness and elasticity, retain the natural color, and be susceptible of the desired polish. The following proportions I find to answer these ends better than any other that I have tried:

| | | | |
|---|---|---|---|
| By weight: | 45 to 50 | parts of | pure gold. |
| " | 2 | " | silver. |
| " | $\frac{3}{4}$ | " | copper. |
| " | $\frac{1}{4}$ | " | platinum. |

I first prepare the alloy as follows: platinum, 1 part; copper, 3 parts; silver, 8 parts, (or from 6 to 8 parts.) This forms a very pretty alloy, of a bright silver color, with a yellow tinge. It is quite hard, and of a firm, close structure: malleable, and bears a fine polish. With this, I alloy the gold, to from $21\frac{1}{2}$ to $22\frac{1}{2}$ carats, according to the fineness required, (by adding $2\frac{1}{2}$ or $1\frac{1}{2}$ parts, as the case may be, to $21\frac{1}{2}$ or $22\frac{1}{2}$ of pure gold.)

It may not be known to all of your readers that platinum can be made to unite *directly* with gold, as also with silver and copper, by the heat of the common blow-pipe. Many dentists are in the habit of filing platinum, in order to melt it with gold, and some are under the belief that, even then, it does not really fuse, but rather *mixes* with the latter, which has frequently led them to throw away their chippings from the rivets of artificial teeth, lest the presence of such should spoil their plate. Although always satisfied in regard to the actual fusion of platinum with gold, yet I confess, laboring under the impression that, at the ordinary heat, this took place only at the surfaces of contact, and that hence it was necessary that the platinum be in a state of minute division as in the form of filings, in which I used it. Nor was this error corrected, until after trying some quite coarse filings with gold, when, finding this to fuse under the blow-pipe, I tried next rivet chippings, and then larger masses of platinum with the same success. The platinum *amalgamates*, as it were, with the fused metal, in the same way that gold combines with melted tin.

All that is necessary, is to put your scraps to be fused into a depression made in a piece of charcoal, and then apply an intense heat by means of the blow-pipe. In alloying platinum with copper, in this way, it is well to cover in part with another coal, that the copper may not oxydize too soon. In this manner, every one can make his own alloy, and then, if he chooses, send the proper proportions to the goldsmith, for melting and rolling.

I have frequently made inquiries of dentists, (whom I supposed likely to be informed on the subject,) in regard to the mode and proportion of mixing platinum with gold, but without obtaining any thing satisfactory; it may have been from a want of the knowledge sought after, or to a disposition to keep it a "*secret.*" In either case, the above hints may not be without value to some in the profession. At any rate, there are many things which, though they should not prove to be *new*, it is well to bring into notice anew, so that they be not forgotten. A case in point, is the mode of "welding" platina, recently communicated to the profession by Dr. J. Allen, of Cincinnati, but which was performed in the time of M. Delabarre. "In order to accomplish it, he placed a very thin gold plate, twenty carats fine, beneath the two leaves of platina, fastened them by two rivets, and soldered them by the fusion of the intermediate leaf."* This is upon the same principal of that published in the *New York Dental Recorder* for February, viz. by *soldering* through the means of gold. And yet Dr. Allen none the less deserves our thanks for suggesting its application, in uniting together platina fragments, otherwise useless for the purposes in which this metal, as a mass, comes into requisition.

*Gold Solder as Fine as the Plate.*—From various experiments, I find the following answers best in point of color, &c., for the above plate; pure *gold*, 22 parts; *tin*, 1 part; *zinc*, 1 part. This, by annealing, is sufficient malleable for solder; melts easily enough, and flows well. It will be seen that *it is twenty-two carats fine.*

* *Desirabode's Science and Art of Dentistry;* Dental Library edition, page 430.

Judging from a series of experiments in making solder with tin, zinc, brass, copper, &c., I am led to believe that copper adds nothing to the fusibility of the alloys, nor assists in making it flow better. It would seem to be the combination of *zinc* that gives the *flow*.

The advantages of this solder are, 1st, its fineness; 2d, its being of the same standard as the plate used, thereby obviating the galvanic action resulting from a difference in fineness of plate and solder; 3d, it is without the copperish taste so frequently experienced from solder alloyed with copper; and 4th, it has not the hardness which this metal communicates, but on the other hand, can be as easily filed or cut, and leveled off, smoothed, and polished, as the plate itself.

I first make an alloy of four parts gold (pure;) one tin, and one zinc. This forms a grayish white, and very brittle mass. Although sixteen carats fine—or as fine as *jewelers' gold*, which some "dentists" use for plate!—it crumbles under the hammer, and melts very readily. Of this, take one part to three of gold, and you have the above.

---

### ON MAKING SOLDER.—CONTINUED.

Messrs. Editors:—In your last number I gave a recipe for making 22 carats fine, for gold plate of the same fineness, by alloying pure gold with equal parts of *zinc* and *tin*. As it has been generally received as "the law," in our profession, that solder must necessarily be a few carats *inferior* in standard to the plate on which it is to be employed, and as some may question whether tin and zinc will combine so as to work well with gold, or rather whether gold will "work" at all in companionship with these "base" metals, I have thought that it might not be out of place to send you a sample, that you may judge for yourselves. It consists of three teeth mounted upon a small thin plate, with clasps attached, all united with the above solder. The plate is 21 carats fine, being pure gold alloyed with equal parts of silver, copper and platinum. The color is perhaps, all that could be desired, as well as the elasticity, &c. Regarding platinum as equal to gold, the plate may pass for 22 carats fine. The solder is 22 carats, made by alloying pure gold, (of the same piece used for the plate,) with equal parts of zinc and tin. It will fuse on gold of an inferior standard, 20 carats certainly, and I think likely as low as 18 or 19. The single tooth I send, that you may try its tenacity. The plate of this is about 20 carats, alloyed with silver and copper.

The solder does not flow so freely and evenly as could be wished, but it is as soft as the plate, and can be readily trimmed down with a scraper—a different proportion might improve it in this particular. By continuing the heat, the zinc and tin rise to the surface and are burned off in a great measure, thus leaving the pieces united by almost pure gold.

Some years ago, (it was in 1846,) I made a very fine solder by the use of tin alone, which, although objectionable in some respects, I have frequently found valuable in others. Rolled in strips it is not nearly so brittle as the above—indeed hardly more so than the gold itself; but it melts harder and does not flow well, except under a heat which would jeopardize the plate if employed in soldering sets of teeth. It fuses *in situ*, as it were, clinging however to the plate with great tenacity. I employed it in two or three cases, in parts of sets without accident, but have chiefly found it useful in uniting strips for clasps, or in thickening the edges of my plate where I wished to prevent the stiffness that would have rendered the latter objectionable. I have no doubt that some of the other base metals might be found serviceable. The subject certainly seems worthy of experimental inquiry, on the part of the dental profession.

If the use of 15 or 16 carat gold is objectionable for *plate*, it should also be so for *solder*. In full sets of teeth as much solder is sometimes used as might suffice to make some of the thin, narrow plates which we frequently find in parts of sets. The salts of copper liable to be formed in the mouth from this metal, as contained in the ordinary solder, might certainly act perniciously in cases where the compounds, produced under similar conditions, from a like proportion of tin or zinc, would be comparatively innocuous; but apart from this, the small proportion required of the latter metals for the above purpose, must obviate all objection on this score.

While writing, I will here take the opportunity of correcting an erroneous impression that may have been conveyed by a general remark in my last, wherein I spoke of having frequently made inquiry of dentists, whom I supposed likely to be informed on the subject, in regard to the use of platinum, without obtaining anything satisfactory, &c. This appears to have been regarded by some, as intended to apply to the profession in this city, thereby conveying the impression abroad that an illiberal spirited existed in our midst. I did not certainly intend this. The members of the profession in this place are not only skillful dentists, but I have ever found liberal minded and free to communicate their "*modus operandi*" in the art. Taking for granted that we all used platinum in nearly the same manner and proportion in alloying plate, and knowing that the plate thus prepared by all, was liable to the objection (in regard to color,) which I sought to remedy, I had not recently conversed with any upon the subject. In alloying gold with platinum, Dr. Gunn and Dr. Ross, have, like myself, been using the latter, in the form of filings. Drs. Hamlin and Morgan, inform me that they have, for some time past, used it in the form of leaf or thin strips. The proportions vary, according to the elasticity required, from say, 2 to 5 grains the dwt. I have tried plate with one-third platinum, but this quantity does not appear to give any greater elasticity than a smaller proportion. An alloy of equal parts of copper, silver and platinum, possesses, perhaps, all the advantages of that which I proposed in your last number.—*Dental News Letter.*

